# Risk factors for venous thromboembolism in metastatic colorectal cancer with contemporary treatment: A SEER‐Medicare analysis

**DOI:** 10.1002/cam4.4581

**Published:** 2022-02-06

**Authors:** Steven Ades, Bhargavi Pulluri, Chris E. Holmes, Inder Lal, Santosh Kumar, Benjamin Littenberg

**Affiliations:** ^1^ University of Vermont Larner College of Medicine Burlington Vermont USA; ^2^ Ascension Saint Agnes Hospital Cancer Institute Baltimore Maryland USA; ^3^ New York Oncology Hematology Amsterdam Cancer Center Amsterdam New York USA; ^4^ Hematology Oncology Associates of CNY East Syracuse New York USA

**Keywords:** bevacizumab, cohort studies, colonic neoplasms, survival analysis, venous thromboembolism

## Abstract

**Background:**

The relationship between metastatic colorectal cancer (mCRC) and venous thromboembolism (VTE) is poorly defined in the modern era. Our objective was to examine impact of putative risk factors including newer treatments and anti‐angiogenic therapy on VTE incidence and survival in a modern older mCRC cohort.

**Methods:**

This is a SEER‐Medicare cohort analysis of mCRC patients diagnosed in 2004–2009. Risk factor analysis was conducted using Cox models adjusted for sex, diagnosis age, race, primary tumor location, comorbidity, and prior VTE history, with cancer treatments as time‐varying covariates. Main outcomes were VTE incidence and overall survival.

**Results:**

Ten thousand nine hundred and seventy six mCRC cases with mean age 77.9 years (range 65–107), 49.7% women, 83.5% white. There were 1306 VTE cases corresponding to 13.7% incidence at 1 year and 20.3% at 3 years. Independent VTE predictors included female sex (HR 1.27; 95% CI 1.14–1.42), African American race (HR 1.49; 1.27–1.73), prior VTE history (HR 16.3; 12.1–22.1), and right sided cancers (HR 1.16; 1.04–1.29). After adjustment, any therapy and bevacizumab (HR 0.68, 0.58–0.78) in particular were protective. Overall survival was 40.1% (39.4–41.3) at 1 year but improved significantly with any treatment. VTE following diagnosis of mCRC was associated with inferior OS (HR 1.09; 1.02–1.15).

**Conclusions:**

In this large contemporary mCRC cohort, effective systemic therapy including anti‐angiogenic treatment was associated with lower VTE risk. Overall survival was poor, and modestly worse if a patient had a VTE at any time during treatment.

## Graphical abstract

In a large contemporary SEER‐Medicare cohort, the cumulative 1‐year incidence remains high at 14%. Current standard therapies for advanced colon cancer including bevacizumab are associated with lower VTE risk and significant survival gains.

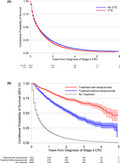

## 1 INTRODUCTION

Colorectal cancer (CRC) is the third most common cancer diagnosed in both men and women in the United States. and the third leading cause of cancer death.^1^ Approximately half of patients diagnosed with CRC develop metastatic disease (mCRC), placing them at higher risk of venous thromboembolic complications.^2^, ^3^ Multiple risk factors increase the risk of VTE in patients with cancer, including cancer‐associated hypercoagulable state, older age, advanced stage, prolonged immobilization, chemotherapy, vessel stasis from direct tumor compression, frequent hospitalization and surgery.^4^, ^5^ VTE causes considerable morbidity and increases the risk of death in the general population even several decades after VTE diagnosis.^6^ However, in the contemporary era of novel systemic therapies, improved VTE treatment, and a shift in management into the outpatient setting, less is known about the effect of anti‐cancer agents on established VTE risk factors and clinical outcomes.^7^

The contemporary management of mCRC has evolved rapidly and involves multiple active drugs, either in combination or as single agents. These include 5‐fluorouracil (5‐FU), capecitabine, irinotecan, oxaliplatin, bevacizumab, epidermal growth factor receptor and tyrosine kinase inhibitors, and checkpoint inhibition in select patients.^8^, ^9^, ^10^, ^11^, ^12^ Cancer‐associated hypercoagulability in combination with cytotoxic chemotherapy increases the risk of VTE.^13^, ^14^ Cisplatin therapy in particular, is a well‐established risk factor for thrombosis, but it is less clear if oxaliplatin poses additional risk.^15^, ^16^, ^17^ Bevacizumab, an inhibitor of vascular endothelial growth factor, was approved for patients with mCRC in 2005,^18^, ^19^ and is associated with increases in arterial and possibly venous thrombotic risk, although the literature is conflicting about the latter.^20^, ^21^, ^22^, ^23^

The validation of risk factors for VTE in an older contemporary mCRC cohort may improve outcome by improving patient selection for effective thromboprophylaxis.^24^, ^25^ Using the linked Surveillance, Epidemiology, and End Results (SEER) and Medicare claims database, we analyzed risk factors associated with VTE in older patients with mCRC in the current treatment era.

## 2 METHODS

The study was conducted using data from the SEER database, which collects and publishes cancer incidence and survival data from population‐based cancer registries covering approximately 28% of the population of the United States. Medicare claims for covered health care services from the time of a person's Medicare eligibility until death are linked, allowing identification of specific treatments received. The SEER database also provides information regarding patient's socio‐demographic status and survival. Given the retrospective cohort design without direct access to personal patient identifiers and no formal consent requirement, institutional review board approval was unnecessary.

### 2.1 Subjects

The cohort included patients with advanced (stage IV) colorectal carcinoma diagnosed from 2004 to 2009 and followed through 2011. The majority of systemic agents available for upfront management of advanced colorectal cancer were approved in (e.g., oxaliplatin, bevacizumab) or prior to 2004 (irinotecan in 2000). Disease diagnosis used the International Classification of Diseases, Ninth Revision, Clinical Modification (ICD‐9‐CM) nomenclature. To minimize bias due to unobserved claims data, we included only patients with both Medicare Part A and Part B coverage for at least 12 months prior and 2 years after diagnosis.

CRC was identified by, primary site using ICD‐O‐3 codes (International Classification of Diseases for Oncology, third edition) and metastases at the time of diagnosis. Only tumors with adenocarcinoma histology were included. Anatomically, tumors were divided into right, left or unknown. Tumors in the cecum, ascending colon, hepatic flexure and transverse colon were considered as right sided colon cancer and tumors in the splenic flexure, descending colon, sigmoid colon and rectum were included on the left sided colon cancer category.

Patients with a history of VTE within 6 months before diagnosis of stage IV colorectal cancer were excluded from final analysis. Patients with another non‐dermatologic malignancy diagnosed before mCRC were excluded.

### 2.2 Outcome

VTE was identified as either deep venous thrombosis (DVT) or pulmonary embolism (PE) using International Classification of Diseases, 9th Revision (ICD‐9) claims codes. A VTE event was defined as either a hospital admission with a primary diagnosis of DVT or PE, or two outpatient services for VTE greater than 7 days apart, but less than 31 days apart. Outpatient services delivered all within a week were not considered VTEs because they probably represented negative diagnostic workups. The initial date of a VTE episode was defined as the earliest date of any outpatient claims or the midpoint of the hospital admission. A VTE event was considered as resolved after 60 consecutive days without any services for VTE. Prior VTE events were treated as unrelated to mCRC, and a potential risk factor for subsequent events.

### 2.3 Risk factors

Potential risk factors including age, race, ethnicity, gender, marital status, date of diagnosis of metastatic colon cancer, tumor location, and prior history of VTE were collected from the SEER data. Geographic SEER registry code city size and state of residence were also selected as rough socioeconomic status determinants.

Comorbidity is an important risk factor in advanced CRC and may contribute to VTE risk as well. The Charlson Comorbidity Index (CCI) represents the weighted number of select comorbid conditions based on ICD codes.^26^ Based on the number of co‐morbidities and resultant left shifted distribution, we classified patients into four categories with CCI of 0, 1, 2 and 3 to 18.

Treatment with chemotherapeutic agents (oxaliplatin, irinotecan, 5‐fluorouracil, capecitabine), anti‐vascular endothelial growth factor inhibitor (anti‐VEGF: bevacizumab), and anti‐epidermal growth factor receptor therapy (anti‐EGFR: cetuximab, panitumumab) were captured as time–time‐varying covariates given that the risk of thrombosis will be temporally associated with administration of the agents and a short time window (1 month selected) following discontinuation.

### 2.4 Statistical analysis

The primary goals of the study were to measure the incidence of VTE and overall survival in a contemporary mCRC cohort, and to identify risk factors, in particular examining the impact of anti‐VEGF and platinum therapy on VTE risk. Kaplan–Meier curves and Cox Proportional Hazards models were used to estimate VTE incidence and mortality and identify independent predictors after controlling for multiple demographic and treatment status variables.^27^ Cases were censored at the time of loss of insurance. Predictors and covariates were allowed to vary over time. For instance, a case may have been exposed to a bevacizumab during some months and not others. These time‐varying analyses allow estimation of effect sizes under conditions where treatment is assigned irregularly, as is common in clinical medicine.^28^ Statistics are reported as point estimates and 95% compatibility^29^ intervals (CI). Analysis was performed using Stata version 14.2.

## 3 RESULTS

**FIGURE 1:**
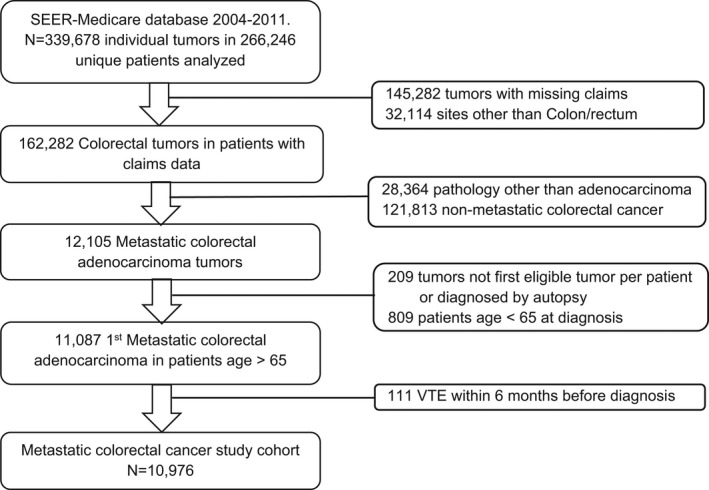
SEER‐Medicare cohort flow diagram

Of 339,678 individual tumors, 10,976 mCRC cases were identified during a 6‐year time period from January 2004 to December 2009 (Figure 1). The mean age in the study sample was 78 years (range 65–107), with 49.7% women, 83.5% white, and 94.4% non‐Hispanic. Right sided tumors were seen in 43.3% of the cases. CCI of 0–1 was seen in 76.9% of the patients, and 1.6% had a history of pre‐existing VTE (Table 1). A plurality of the records was from the California SEER database (29%), followed by New Jersey (16%) and Georgia (10%). Histologic type by ICD‐O‐3 code was “Adenocarcinoma, NOS” in 92% of cases, with the remainder being adenocarcinoma arising in adenomas.

**TABLE 1:** Demographic, cancer characteristics and comorbidity by venous thromboembolism (VTE) diagnosis

Variable	Patients without VTE*n* (%)	Patients with VTE*n* (%)	Total	*p* value^b^
Total	9670	1306	10,976	
Age				
Mean	78.1	76.6	77.9	<0.0001
Range	65–107	65–95	65–107	
Race^a^				
White	8085 (88)	1080 (12)	9165	<0.001
African American	1081 (85)	196 (15)	1277	
Other	504 (94)	30 (6)	534	
Ethnicity				
Non‐Hispanic	9122 (88)	1238 (12)	10,360	0.498
Hispanic	548 (89)	68 (11)	616	
Gender				
Male	4919 (89)	607 (11)	5526	0.003
Female	4751 (87)	699 (13)	5450	
Married				
Yes	4559 (88)	648 (12)	5207	0.069
No	4779 (89)	609 (11)	5388	
Side of the tumor				
Right	4156 (87)	599 (13)	4755	0.056
Left	4820 (88)	631 (12)	5451	
Unknown	694 (90)	76 (10)	770	
Prior VTE				
Yes	129 (74)	46 (26)	175	<0.001
No	9541 (88)	1260 (12)	10,801	
Charlson Index				
0	5809 (87)	860 (13)	6669	<0.001
1	1575 (89)	193 (11)	1768	
2	845 (89)	101 (11)	946	
3	1441 (90)	152 (10)	1593	
City of residence size				
>1 million	5164 (87)	799 (13)	5963	<0.0001
<1 million	4506 (90)	507 (10)	5013	

Abbreviation: VTE, venous thromboembolism.

^a^ In order to comply with CMS cell size suppression policy, Native American race was collapsed into the Other category, along with Asian which was the predominant race in that category.

^b^ Based on Chi‐square testing for categorical variables and *t*‐test for continuous variables.

**FIGURE 2:**
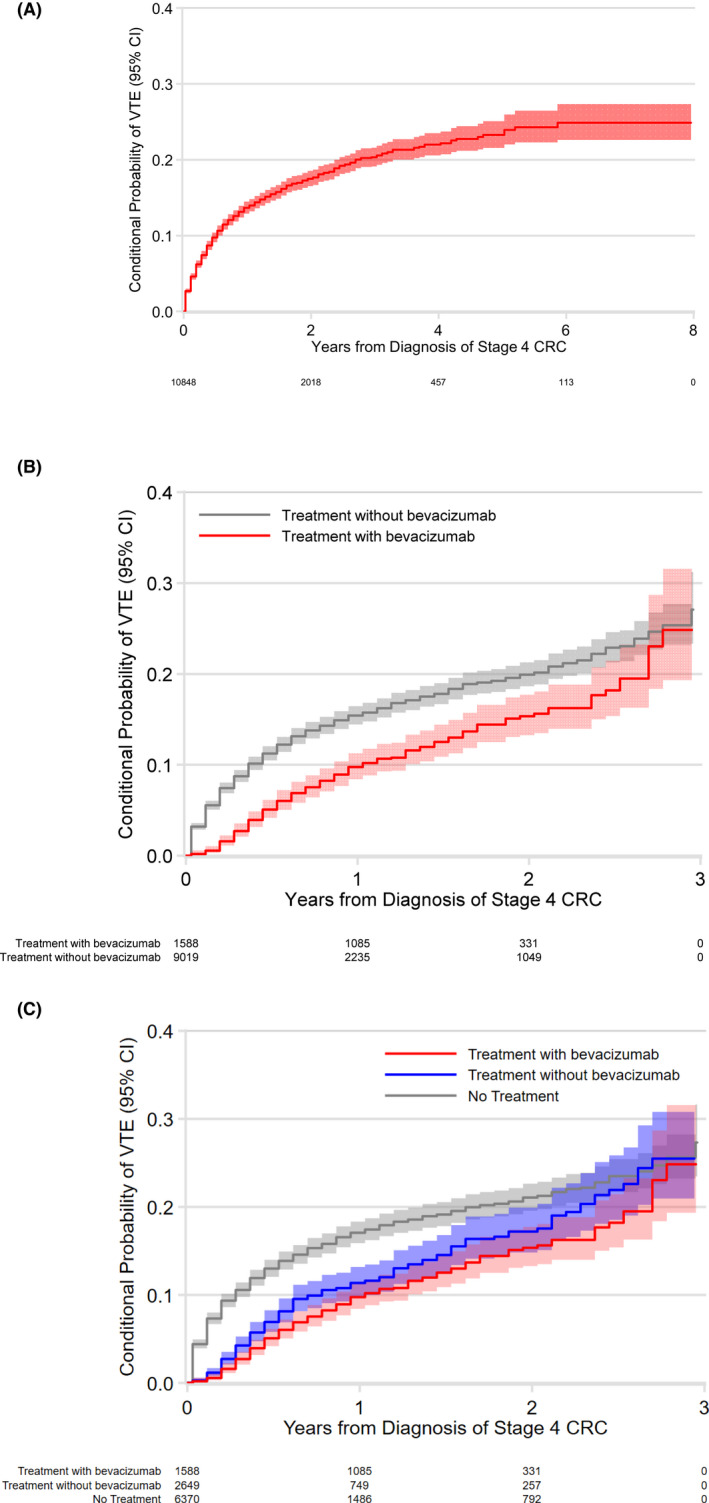
VTE incidence^a^ in (A). overall cohort, (B). according to bevacizumab treatment status^b^, and (C). according to chemotherapy treatment status with or without bevacizumab^b^. CI, confidence interval; CRC, colorectal cancer; VTE, venous thromboembolism. ^a^Kaplan Meier analysis censoring for insurance coverage loss. Treatments vary over time within patient. ^b^Wilcoxon test *p* = <0.001

Following the diagnosis of mCRC, the median VTE‐free survival was 7 months (range 0.5–98 months) in those who did not develop a VTE, and 3.5 months (range 0.5–78 months) in those with VTE. During this time period 1306 cases (11.8%) had VTE with a cumulative incidence of 13.7% (95% CI 12.9–14.5) at 1 year, and 20.3% (95% CI 19.1–21.6) at 3 years (Figure 2A). Table 1 shows the distribution of socio‐demographic and clinical characteristics by VTE diagnosis. On univariate analysis, significant predictors of VTE included younger age, black race, female gender, lower CCI, prior VTE history and larger city of residence (Table 1). There was no impact of calendar year of diagnosis on VTE incidence within the study period. On univariate analysis, VTE incidence was associated with geographic SEER registry code and was highest in Michigan (16.0%) and New Jersey (15.9%), and lowest in Hawaii (6.2%).

From the total cohort of 10,976 mCRC patients, 46.1% received treatment (Table 2). Of these, 86.4% received chemotherapy, and 56.2% received bevacizumab. The most common chemotherapeutic agents used were 5‐FU (34.3%), oxaliplatin (26.2%), and irinotecan (18.1%). Anti‐EGFR therapy was used in 9.1% of patients. On univariate analysis, VTE incidence was higher after any anti‐cancer treatment except capecitabine (Table 2).

**TABLE 2:** Treatment received by venous thromboembolism diagnosis

Variable	Patients without VTE*n* (%)	Patients diagnosed with VTE*n* (%)	Total	*p* value^a^
*Treatment received*				
Yes				
Chemotherapy^b^	3661 (84)	716 (16)	4377	<0.001
5‐FU	3131 (83)	632 (17)	3763	<0.001
Oxaliplatin	2387 (83)	485 (17)	2872	<0.001
Irinotecan	1637 (82)	351 (18)	1988	<0.001
Capecitabine	144 (87)	21 (13)	165	0.74
Bevacizumab	2374 (84)	469 (17)	2843	<0.001
No				
Chemotherapy^b^	6009 (91)	590 (9)	6599	
5‐FU	6539 (91)	674 (9)	7213	
Oxaliplatin	7283 (90)	821 (10)	8104	
Irinotecan	8033 (89)	955 (11)	8988	
Capecitabine	9526 (88)	1285 (12)	10,811	
Bevacizumab	7296 (90)	837 (10)	8133	

Abbreviation: VTE, venous thromboembolism.

^a^ Univariate Chi‐square analysis for comparison of VTE rate according to receipt of each different anticancer agent.

^b^ Includes 5‐FU, oxaliplatin, irinotecan and capecitabine.

In multivariate models, significant predictors of VTE included female gender (HR 1.27; CI 1.14–1.42), African American race (HR 1.49; CI 1.27–1.73), Asian race (HR 0.45; CI 0.31–0.67), prior history of VTE (HR 16.3; CI 12.1–22.1), right sided colon cancers (HR 1.16; CI 1.04–1.29), and higher CCI (HR 0.96; CI 0.92–0.99). After adjustment, any systemic treatment was protective (Figures 2B,C). Bevacizumab was particularly protective (HR 0.68, CI 0.58–0.78) in comparison to patients receiving no therapy. VTE incidence at 2 years post‐diagnosis was 14.6% (CI 12.7–16.8) with bevacizumab therapy compared to 18.0% (CI 16.9–19.2) without it. Compared to untreated patients, oxaliplatin therapy was also protective (HR 0.73; CI 0.63–0.85), as was non‐bevacizumab‐containing systemic therapy (HR 0.64; CI 0.55–0.74) and systemic treatment in combination with bevacizumab (HR 0.60, 0.52–0.70).

**FIGURE 3:**
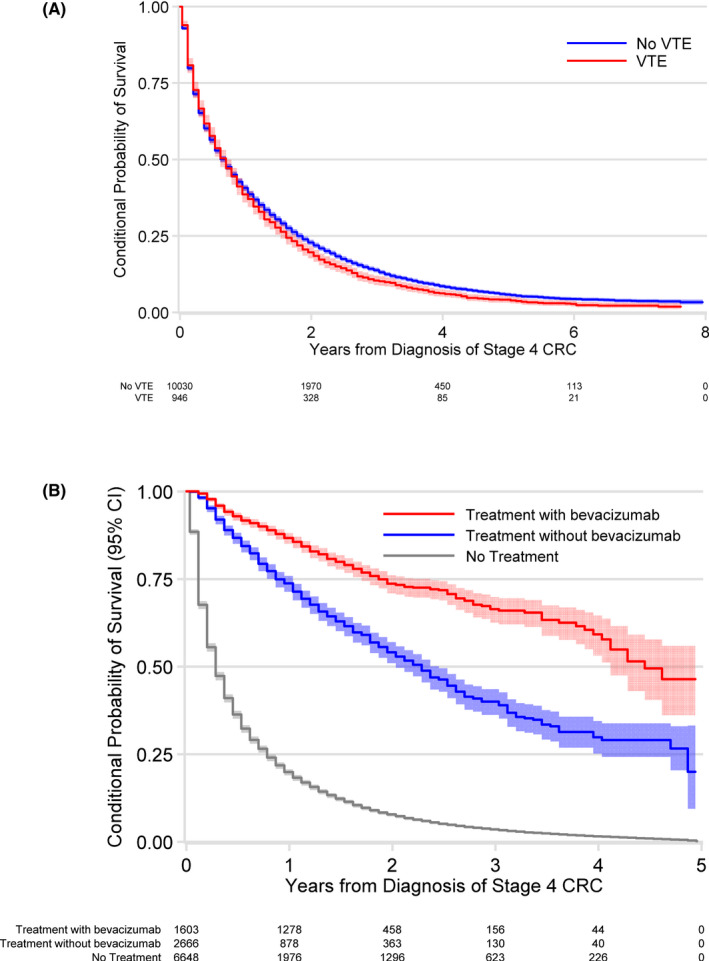
Overall survival^a^ in (A). According to VTE status, and (B). According to chemotherapy treatment status with or without bevacizumab^b^. CI, confidence interval; CRC, colorectal cancer; VTE, venous thromboembolism. ^a^Kaplan Meier analysis censoring for insurance coverage loss. Treatments vary over time within patient. ^b^Wilcoxon test *p* = <0.001

Overall survival (OS) was poor with only 40.1% (CI 39.4–41.3) and 13.3% (CI 12.6–13.9) alive at 1 and 3 years, respectively. Adjusting for other covariates, VTE following diagnosis of mCRC was associated with reduced OS (HR 1.09; CI 1.02–1.15) (Figure 3A). However, median OS was 8 months, and identical in patients with or without VTE. Other independent predictors of poor OS included age (HR 1.34; CI 1.31–1.38), higher CCI (HR 1.06; CI 1.04–1.07), African American race (HR 1.19; CI 1.12–1.27), and right‐sided tumor location (HR 1.08, CI 1.04–1.12). OS was significantly improved with therapy, with a median survival of 36 compared to under 6 months. Among the patients who received therapy, the combination of chemotherapy and bevacizumab was associated with superior overall survival compared to patients who received only chemotherapy (Figure 3B).

## 4 DISCUSSION

While prior research has established the link between CRC and VTE, it generally focused on VTE incidence and inpatient treatment in the post‐operative setting, earlier disease stages, trial populations, or older registry cohorts preceding the contemporary treatment era^30^, ^31^, ^32^ with introduction of oxaliplatin, irinotecan and bevacizumab. The primary aims of this study were to measure the incidence of VTE and its impact on survival, as well as risk factors for both VTE and OS in a mCRC patient population with particular focus on newer anti‐angiogenic and platinum therapies.

Our analysis, which captured both inpatient and outpatient VTE events, reported a 20% risk of VTE over 3 years post‐diagnosis, almost half of which occurs in the first year of mCRC. This risk of VTE, particularly in the first year of therapy, is consistent with prior observations. For example, a retrospective study reported an annual incidence of 10.9% of VTE in CRC patients treated with chemotherapy, with a higher incidence of 15% in association with 5FU treatment.^33^ In contrast, other studies observed lower event rates including a Californian cohort trial reporting a 2‐year cumulative incidence of only 4.7% in the mCRC setting,^30^ and a British cohort trial that reported an incidence rate of 41.3 per 1000 person‐years, translating into an incidence estimate of 9.1% with a 2.2‐year median follow‐up.^34^

This is first the publication to our knowledge which has shown a link between tumor‐sidedness with both VTE risk and survival. Recent publications have highlighted the importance of the right sided tumor location as a predictor for poor OS, but fail to fully explain the etiology behind this association.^35^, ^36^, ^37^ Clinically, right‐sided tumors are more commonly associated with poor prognostic indicators such as RAS and BRAF mutations, microsatellite instability, CpG island methylator phenotype, and mucinous histology.^38^ Mucin‐secreting adenocarcinomas in particular, are frequently associated with thrombosis, which may partly explain the association between sidedness of CRC and VTE risk.^39^

Important VTE risk factors in this analysis included race, location of primary tumor, and antecedent history of VTE. In agreement with prior CRC studies, African American patients had higher and Asian patients had lower VTE risk compared to white patients.^30^, ^40^ African American race was also an independent predictor for mortality in this setting.

In multivariate analysis, VTE preceding the diagnosis of mCRC by 6 months or greater was the strongest predictor of subsequent VTE, although only 1.6% of patients had a prior VTE. Prior history of VTE is an established risk factor for future VTE in the general population, hospitalized patients, and patients undergoing cancer surgery,^41^, ^42^ with growing evidence supporting an association with VTE in ambulatory cancer patients on treatment beyond traditional risk models such as the Khorana score.^43^

After adjustment for independent baseline predictors, both chemotherapy and anti‐angiogenic treatment with bevacizumab were associated with lower VTE incidence and improved survival, consistent with recent trial estimates.^18^, ^44^ The decrease in VTE in treated patients is consistent with disease control as a result of effective therapeutic options. Bevacizumab is recognized as a predictor for arterial thromboembolic events, but its role in VTE is controversial.^20^, ^45^, ^46^ The incidence of VTE in bevacizumab‐treated patients varies substantially across tumor types, from 3% to 19%.^22^, ^47^ A recent meta‐analysis reported a significant association between bevacizumab therapy and VTE risk^23^ in various cancers. Although CRC patients in that analysis had the highest overall incidence of VTE (19.1%), the relative risk was lowest at 1.19 (95% CI 0.92–1.55) with bevacizumab treatment.^23^ A large single‐institution mCRC cohort study failed to show any relationship between bevacizumab and VTE,^20^ while a population‐based mCRC cohort trial identified high cumulative exposure to bevacizumab as an independent risk factor.^46^ Our data also fails to show a relationship between bevacizumab therapy and VTE in a large representative contemporary mCRC cohort, after adjustment for other systemic treatment.

Despite a meaningful survival advantage with treatment, median OS remains poor in this cohort. DVT is associated with a modest negative impact on OS. Higher CCI was associated with increased mortality, and the lower risk of VTE in patients with higher comorbidity suggests the effect of competing mortality, as these patients may die before developing VTE.

Limitations of this study include those inherent to a retrospective observational administrative database with potential for misclassification of predictors, outcomes and covariates. Important covariates such as body mass index and laboratory test results which contribute to the Khorana risk score were unavailable.^48^ We did not control for established risk factors for VTE such as hospitalization or surgery, or directly for socioeconomic status. Immobilization could not be accurately captured in the database either despite the use of a score combining billing for either an assist device or supplemental oxygen as a surrogate for functional assessment. Information bias may affect prior VTE event estimates. This study did not control for prophylaxis, although it would have been rare outside of inpatient or perioperative settings. Sources of missing information also include care delivered elsewhere, such as veteran's hospitals and free clinics. Finally, older patients with mCRC residing in the SEER geographic areas may differ from patients residing elsewhere in the United States.

## 5 CONCLUSION

VTE remains an important complication in patients with metastatic colorectal cancer with an estimated incidence of 14% at 1 year following diagnosis. Modern therapeutic combinations including anti‐angiogenic or platinum‐based therapy favorably impacted VTE incidence and survival. Poor OS for the entire cohort is consistent with the fact that over half of patients did not receive any systemic treatment. VTE occurrence had only a modest negative impact on survival. The finding that right sided colon cancers were associated with an increased risk of VTE may refine our ability to risk stratify patients in the future. While important risk factors for VTE include race, tumor site, and prior history of VTE, this analysis supports the use of effective systemic therapy as having the greatest positive impact on VTE incidence and OS.

## CONFLICT OF INTEREST

There are no relevant COI disclosures for any of the contributing authors.

## AUTHOR CONTRIBUTIONS

All authors contributed to substantively to study design, analysis, and interpretation of this study. SA drafted the manuscript and revisions, but all authors reviewed it for accuracy.

## Data Availability

The data that support the findings of this study are available from SEER‐Medicare. Restrictions apply to the availability of these data, which were used under license for this study. Data are available from the corresponding author with the permission of SEER Program IMS.
